# Diseases of the Nervous System

**Published:** 1894-05-26

**Authors:** 


### Medical Progress.

### DISEASES OF THE NERVOUS SYSTEM.

*Chorea.*—Charles L. Dana[1] gives a detailed account of an important case bearing upon the etiology and pathology of Sydenham's chorea, in continuation of the views he expressed in Brain[2] as to the essential seat and cause of the disease. The patient was a man of thirty-four, who, having had acute rheumatism at ten years of age, became choreic at fourteen and had continued so with slight intermissions until the time of his death. At the autopsy the dura mater was normal; over the convexity of hemispheres on both sides was a granular thickening of the pia mater and arachnoid, presenting the appearance of a chronic proliferative meningitis. This extended from the base of the first frontal convolution backwards to near the occipital lobe; it extended downward nearly half-way upon the great convexity of the brain and about half-way also upon the surface within the median fissure. On cutting through the membrane it was found to be thickened and attached to the surface beneath. Upon microscopical examination of the thus affected area the pia mater was found to be thickened by connective tissue proliferation, but without exudation or round-cell infiltration. The superficial or neuroglia layer of the cortex showed distinct softening and degeneration with a rich cellular infiltration apparently coming from the meninges, the infiltration not extending beyond the neuroglia layer. Numerous hyaline masses were seen both in the pia mater and just below it extending in small numbers as far as the second layer of the cortex. It was in the region where these degenerative changes were seen that the micro-organisms were found. The nerve cells proper did not show very decided changes, the hyaline masses being derived from degeneration of neuroglia or pyramidal cells. The bacteriological examination by Dr. H. T. Brooks, revealed a small diploccocus confined in locality to the inner surface of the membrane, and in that portion of the cortex immediately below the junction with the meninges, a small diplococcus resembling in every detail that present in the brain covering was seen. The micro-organisms were about half the diameter of a red blood cell, and resembled very closely the diplococcus lanceolatus; although not in large numbers, they were found in all the sections, and always in the deeper layers of the pia and superficial part of the cortex. Dr. Dana concludes that this case confirms the view that chorea is a vascular and humoral disease; that in some cases at least there is a microbe which produces the disease; that the seat of the lesion is either meningeal or superficially cortical in the brain; as regards the spinal cord, the seat of the lesion is mainly in the meninges and blood vessels, where it surrounds and irritates the roots of the nerves, and finally, that chorea cannot be explained by finding any particular seat of the disease. Chorea has not any special form of anatomical change associated with it, though degenerative hyaline change and evidences of vascular irritation are common. In order to produce chorea there must be a specific kind of irritation of the cells; this may be a rheumatic poison or diplococcus toxin, but the specific irritants are not numerous, as chorea occurs but rarely, and only when the proper regions are properly irritated, notwithstanding the many forms of injury and disease to which the motor fields are subject. The various types of chronic chorea would be explained by the variable intensity of the irritation, its special localisation, and the degree of organic change eventually induced. There is nothing which can so well explain the phenomena of the disease as to suppose that the specific agent producing chorea is a microbe, and perhaps some form of diplococcus.

Upon the pathology of hereditary (or Huntingdon's) chorea Oppenheim and Hoppe[3] give the result of the microscopical examination of two cases. In both there was a certain amount of atrophy of the cerebral convolutions associated with some external hydrocephalus. Many microscopic alterations in the nervous system were found, but the authors consider that the essential one of these was a miliary disseminated encephalitis of the cortical and sub-cortical zones, especially in the motor regions. The inflammation may go on to sclerosis, and lead to cortical atrophy, and sometimes to external hydrocephalus. Some comparatively trivial changes, which took their origin in the neuroglia and blood-vessels were found in the spinal cord. The authors consider that the degeneration in the nerves and changes in the muscles are not necessary features of the disease.

*Cortical Localisation.*—(*a*) *Centre of Movements of the Face.*—Brissaud[4] reports a case in which there had existed paralysis of the right side of the face (the permanent residue of an original right hemiplegia and aphasia) for two years before death. At the autopsy there was found a single cortical lesion—a yellow softening in the region of the left Rolandic operculum immediately behind the frontal operculum, that is, immediately behind the lower end of the fissure of Rolando. The softening extended to the upper sulcus of the Island of Reil. Considering the very small focus of the lesion, the deduction from this study of the centre for face movements in man was unique.

(*b*) *Localisation of Pure Word-Blindness.*—Déjèrine and Vialet[5] record the case of a cultured man in whom there existed absolute verbal blindness, both for letter and words, as well as musical blindness, but the ability to read figures and to calculate was preserved. There was no sign of verbal deafness, and no indication of difficulty in articulate speech. There was no mind-blindness, and no visual aphasia. The power of mimicry was retained, as also was the ability to write spontaneously and upon dictation, but transcription was difficult and imperfect. These symptoms had been present for four years, when death occurred suddenly, paraphasia and total agraphia having existed for two days without a sign of verbal deafness or impairment of general intelligence. At the autopsy an area of recent red softening was found in the inferior parietal convolution and angular gyrus of the left cerebral hemisphere; while areas of old atrophic lesions were found in the lingual lobule, the fusiform lobule, the cuneus and the apex of the occipital lobe, with secondary degeneration in the corpus callosum, and pronounced atrophy in the optic radiations. The right hemisphere was intact. Upon microscopic examination all the white matter of the posterior portion of the left lingual and fusiform lobules, and of the collateral fissure was found to be destroyed and replaced by cicatricial tissue. The lingual lobe was similarly affected to a less degree. The optic radiations of Gratiolet and the inferior longitudinal fasciculus of Burdach were entirely destroyed, and all the structures in the descending branch of the calcarine fissure participated in the softening. From this case the deduction is drawn that the lower portion of the inferior longitudinal fasciculus of Burdach contains physiologically differentiated fibres that connect the visual zone with the zone of language. Hoisholt[6] also records a case of word-blindness and music-blindness without agraphia. Towards the end of the case, however, there was also left homonymous hemianopsia, the visual defect became more pronounced, and the fields of vision more and more contracted until there was total blindness. There was also some deafness. Upon post-mortem examination the whole of the occipital lobe of the left side of the brain was found to be depressed below the general level, presented a yellowish green colour, and the convolutions were reduced in size, these changes extending forward into the angular and supra-marginal gyri, and along the median surface of the occipital lobe. The posterior part of the right hemisphere was also affected by flattening of the convolutions. This case, therefore, in the conclusions to be deduced from it, does not assist in the detailed localisation of the faculty of word-vision, as does the preceding case, but simply shows the general areas in both hemispheres which are concerned in the faculty of vision as a whole.

*Caisson Disease.*—Very interesting cases of this singular disease are reported by Gilman, Thompson, and Andrew H. Smith,[7] with suggestive comments upon the cause of the condition. Men who have been working some few hours in a caisson under a pressure of from two and a-half to four atmospheres are seized. if their return to normal atmospheric pressure is unduly rapid, with a kind of paraplegia. The most constant and prominent of the symptoms are referable for their origin to the inferior portion of the spinal cord. These symptoms are—intense pain in the peripheral nerves, joints, and long bones, especially those of the inferior extremities, various sensory disturbances, altered reflexes, interferences with normal processes of micturition and defæcation, and paralysis of the extremities; there is often also very great pain referred to the abdomen attended with nausea; less frequently there are symptoms referable to the brain, such as deafness, vertigo, and pains in the head. These symptoms, which come on a few minutes after quitting the caisson, generally last a few days only, rarely a few weeks, and still more rarely are fatal or permanent in part. A similar condition is seen in divers occasionally, but in them the abdominal pain seems to be more usually, and the paraplegic symptoms less commonly seen. Some difference of opinion exists as to the cause of this condition. François suggested that they might be due to the liberation of bubbles of air from the blood when the pressure was reduced, the bubbles acting as emboli. Paul Bert maintaining this view asserts that the bubbles are of nitrogen, although his own experiments upon animals showed that a pressure of five atmospheres was required to produce the symptoms, and three minutes spent in graduating the return to normal pressure sufficed to prevent their occurrence—circumstances which do not obtain in these examples of caisson disease, in which also toleration can conspicuously be established. Under the abnormally great atmospheric pressure the blood is forced out of the skin and superficial tissues (causing the waxy pallor of complexion seen in the men while in the caisson) into other tissues which are more protected from such pressure by the rigidity of superjacent structures; this protection is most complete in the bony cavities such as the spinal canal and shafts of the long bones, whither in consequence the flow of blood is determined immensely in excess of the normal amount. The blood vessels in the bony cavities being thus over-distended are reduced to a state of paresis, if not paralysis, so that when, on return of the subject to normal atmospheric pressure the other blood vessels again receive the ordinary blood supply, the vascular tension being thus again lowered, these paretic vessels are unable to relieve themselves of their contained blood. Under the greatly diminished *vis a tergo* this blood ceases to circulate, and the vessels become the seat of thrombosis, with possibly subsequent emboli, evenif hæmorrhage has not already occurred during the period of high tension. This vasomotor theory of explanation is made the more probable, firstly, because ergot is found by Dr. A. H. Smith to be more effectual than morphine in procuring relief; and secondly, that in the few instances in which a post-mortem examination has been obtained soon after the first onset of symptoms, the lower part of the spinal cord is found to be the seat of congestion, and occasionally of of hæmorrhage also. The theory of emboli caused by gaseous bubbles is based upon *a priori* considerations only.

*Hysteria.*—J. K. Mitchell and G. de Schweinitz[8] in an elaborate article record their recent investigations into the fields of vision in hysterical subjects. Nearly thirty years ago Galezowski called attention to the alteration of the colour-sense in this affection, and since then much has been published upon the subject of a rather contradictory nature. The main conclusions arrived at by the aforesaid investigators are: (1) Achromatopsia was not found to be present, as described by Galezowski and French observers. (2) Reversal in the normal sequence of colours (so that red is the largest field) is usually present where there is anæsthesia, but disturbance of colour-sense and anæsthesia are not necessarily accompanied one by the other. (3) The green field is more often contracted than the others. (4) Disturbance of the colour-sense is much more frequent in truly hysteric than in simply neurasthenic patients. (5) The degree of the hysterical manifestations bears no relation to the disturbance of colour-sense. Practically normal fields are to be found where the general symptoms of hysteria—anæsthesia excepted—are of the highest grade. (6) Some of the following changes are likely to be present in cases of hysteria: (*a*) Simple contraction of the colour-fields, form fields being unaffected; (*b*) contraction of both form and colour fields, the green field being relatively the most contracted; (*c*) partial or complete reversal of the normal sequence in which colours are appreciated, the red field being most commonly the greatest in extent. (*d*) Obscurations of portions of the visual fields as hemianopia, or greater contraction of the field on one side than on the other, the greater contraction being usually on the same side as the anæsthesia. From these deductions it is clear that the affection of the fields of vision is variable in kind and of uncertain existence.

An important case is recorded by Leo[9] of death from laryngeal spasm in hysteria. The subject was a man twenty-one years of age, who had for long exhibited various hysterical symptoms, such as areas of anæsthesia, choreiform movements, and others. In a recurrent attack of convulsion, marked by intense inspiratory dyspnœa, singultus and deep cyanosis, the attempt to maintain respiration artificially failed, and death took place. Upon post-mortem examination the brain and membranes were found to be normal. The larynx contained no foreign body and was not œdematous, but the vocal chords were firmly adducted so that water could not pass the rima. The hysterical nature of the affection was recognised during life. It is important to bear in mind that a fatal termination may by possibility occur in hysteria from inspiratory dyspnœa, just as somewhat less rarely we see it in chorea, and hysterical convulsion must not be considered as in its nature to be devoid of serious import.

[1] Amer. Journ. Med. Sci., Jan., 1894. [2] Brain, April, 1890. [3] Archiv. für Pysch., xxv., quoted in Ep. B. M. J., Feb. 24, 1894. [4] Prog. Med., Dec. 30, 1893. [5] Compt. Rend. des Séances de la Soc. de Biol., quoted in Amer. Journ. Med. Sci., Dec., 1893. [6] Occ. Med. Times, Vol. xii., No. 9, quoted in Amer. Journ. Med. Sci., Feb., 1894. [7] Med. Rec., Feb. 3, 1894. [8] Journ. Nerv. and Ment. Dis., Jan., 1894. [9] Deut. Med. Woch., No. 34, 1893.

